# Phycodnavirus Potassium Ion Channel Proteins Question the Virus Molecular Piracy Hypothesis

**DOI:** 10.1371/journal.pone.0038826

**Published:** 2012-06-07

**Authors:** Kay Hamacher, Timo Greiner, Hiroyuki Ogata, James L. Van Etten, Manuela Gebhardt, Luis P. Villarreal, Cristian Cosentino, Anna Moroni, Gerhard Thiel

**Affiliations:** 1 Computational Biology Group, Technische Universität Darmstadt, Darmstadt, Germany; 2 Membrane Biophysics Group, Technische Universität Darmstadt, Darmstadt, Germany; 3 Structural and Genomic Information Laboratory, Aix-Marseille University, Marseille, France; 4 Department of Plant Pathology and Nebraska Center for Virology, University of Nebraska, Lincoln, Nebraska, United States of America; 5 Center of Virus Research, University of California Irvine, Irvine, California, United States of America; 6 Department of Biology, Università degli Studi di Milano, Milan, Italy; Rutgers University, United States of America

## Abstract

Phycodnaviruses are large dsDNA, algal-infecting viruses that encode many genes with homologs in prokaryotes and eukaryotes. Among the viral gene products are the smallest proteins known to form functional K^+^ channels. To determine if these viral K^+^ channels are the product of molecular piracy from their hosts, we compared the sequences of the K^+^ channel pore modules from seven phycodnaviruses to the K^+^ channels from *Chlorella variabilis* and *Ectocarpus siliculosus*, whose genomes have recently been sequenced. *C. variabilis* is the host for two of the viruses PBCV-1 and NY-2A and *E. siliculosus* is the host for the virus EsV-1. Systematic phylogenetic analyses consistently indicate that the viral K^+^ channels are not related to any lineage of the host channel homologs and that they are more closely related to each other than to their host homologs. A consensus sequence of the viral channels resembles a protein of unknown function from a proteobacterium. However, the bacterial protein lacks the consensus motif of all K^+^ channels and it does not form a functional channel in yeast, suggesting that the viral channels did not come from a proteobacterium. Collectively, our results indicate that the viruses did not acquire their K^+^ channel-encoding genes from their current algal hosts by gene transfer; thus alternative explanations are required. One possibility is that the viral genes arose from ancient organisms, which served as their hosts before the viruses developed their current host specificity. Alternatively the viral proteins could be the origin of K^+^ channels in algae and perhaps even all cellular organisms.

## Introduction

In recent years several virus-encoded proteins with ion channel activity have been described [1]–[4]. These proteins show few common features at the sequence level, except that most of them are short, approximately 100 amino acid residues, and their membrane-spanning domains are predicted to be α-helices [1]. The majority of these viral-encoded channel proteins have no recognizable sequence similarity to bacterial or eukaryotic proteins. One exception is the channel forming protein Vpu from the Human immunodeficiency virus type 1 (HIV-1), which slightly resembles the first transmembrane domain of eukaryotic TASK channels; thus this gene might have been acquired from its host via molecular piracy [5].

A different situation occurs with ion channel proteins encoded by the virus family *Phycodnaviridae*. These viruses, which infect algae [6], have gene products with the structural and functional hallmarks of eukaryotic and prokaryotic K^+^ channels [4]. The best-studied viral K^+^ channel is Kcv from *Paramecium bursaria* chlorella virus 1 (PBCV-1) (genus *Chlorovirus*) [7]. Like complex eukaryotic channels this channel functions as a tetramer [8], [9]. Compared to other K^+^ channel proteins, the monomer is small, consisting of only 94 amino acid residues [4], [7]. The monomer forms a structure with two transmembrane domains, which are linked by a pore helix including a selectivity filter [10] present in all known K^+^ channels [7]. Hence, Kcv essentially corresponds to the pore module part of larger K^+^ channels. Kcv has the basic properties of K^+^ channels such as ion selectivity, gating and sensitivity to blockers [7], [8], [9], [11]. Circumstantial evidence suggests that an active Kcv channel is required for PBCV-1 infection [4], [12]. The channel is probably located in the internal membrane of the virus particle. During the early phase of infection the viral internal membrane presumably fuses with the host plasma membrane. This fusion process initiates rapid depolarization of the host plasma membrane [13], which results in a rapid loss of K-salt from the host [14]. As a consequence the internal turgor pressure of the host alga decreases, which makes it easier for the virus to eject its DNA into the host cell [4], [15].

Subsequently, K^+^ channels have been discovered in three other members of the *Phycodnaviridae* that infect different hosts [16]–[22]. Like PBCV-1, these viruses [e.g. *Acanthocystis turfacea* chlorella virus 1 (ATCV-1) and chlorovirus MT325] infect different algal species with a strict host specificity [20]–[21]. Although, the K^+^ channels in these viruses are similar to PBCV-1 Kcv (Kcv_PBCV-1_), they have major structural differences. The most obvious difference is their size as well as the organization of their cytoplasmic domains [4]. Differences also exist in their physiological properties when expressed in heterologous systems. For example, Kcv_PBCV-1_ has a much lower open probability than its homolog from ATCV-1 (Kcv_ATCV-1_). Also, Kcv_PBCV-1_ conducts rubidium (Rb^+^) better than K^+^ whereas the situation is reversed in Kcv_ATCV-1_ [18].

Another K^+^ channel protein, Kesv, is encoded by *Ectocarpus siliculosus* virus 1 (EsV-1), also a member of the *Phycodnaviridae* family, but distantly related to the chloroviruses [23], [24]. EsV-1 has a different life cycle than the chloroviruses; it infects the marine filamentous brown macro-alga *Ectocarpus siliculosus* and it has a lysogenic life cycle. The chloroviruses are lytic and infect unicellular fresh water green algae [23], [25]. Chlorella (Viridiplantae, Chlorophyta) and *Ectocarpus* (Stramenopiles) are distantly related [26] and their last common ancestor probably dates back 500 million years [27].

The chloroviruses and the Ectocarpus virus are not closely related although they both have large genomes of 280 to 370 Kb [23]–[25]. The prototype chlorovirus PBCV-1 has ∼405 protein encoding sequences (CDS), approximately 35% of them encode proteins of known function. A genome comparison indicates that only 10% of the proteins are shared between PBCV-1 and EsV-1 [23]. Among their common gene products is a K^+^ channel protein [16]. The EsV-1 channel protein, Kesv, is slightly larger (124 residues) than those from the chloroviruses [28], [29]. On a sequence basis, however, Kesv resembles the chlorovirus channel proteins and under certain conditions is functional in heterologous expression systems [29]. The major difference between Kesv and the Kcv channels is the sorting of the proteins within cells [29]. In heterologous expression systems the Kcv channels sort into the secretory pathway and finally move to the plasma membrane. In contrast, the Kesv channel is targeted to the mitochondria. This difference in sorting probably reflects different functional roles of these channels due to the different lifestyles of the viruses.

These findings prompted us to examine the origin and evolution of the viral K^+^ channel proteins and the hypothesis that viruses acquire genes from their hosts. The fact that K^+^ channels from all eukaryotes contain a common pore structure that resembles the viral K^+^ channels is consistent with the traditional assumption that viruses are mere ‘gene pick pockets’ [30] and frequently acquire genes from their host via molecular piracy. If the viral channel proteins are simplified versions of cellular proteins recently acquired from their hosts, then we would expect to see a high level of sequence similarity between the viral and host homologs. However, this traditional view of virus evolution has been challenged by recent phylogenetic studies of genes in large eukaryotic viruses and prokaryotic viruses [31], [32]. Comparative genomics studies further suggest that virus evolution can best be understood in terms of reticulated ‘trees’ and mosaic evolution [33]. This means that large DNA viruses fundamentally have a network-based history that does not trace back to a single gene or set of genes. Hence their ancestor probably exchanged vast pools of genetic elements horizontally and generated a reticulated network of genes at an early stage of their evolution. This view is consistent with the genetics of phycodnavirus evolution as these viruses have both prokaryotic and eukaryotic homologs in addition to many other genes with no cellular homologs.

**Figure 1:**
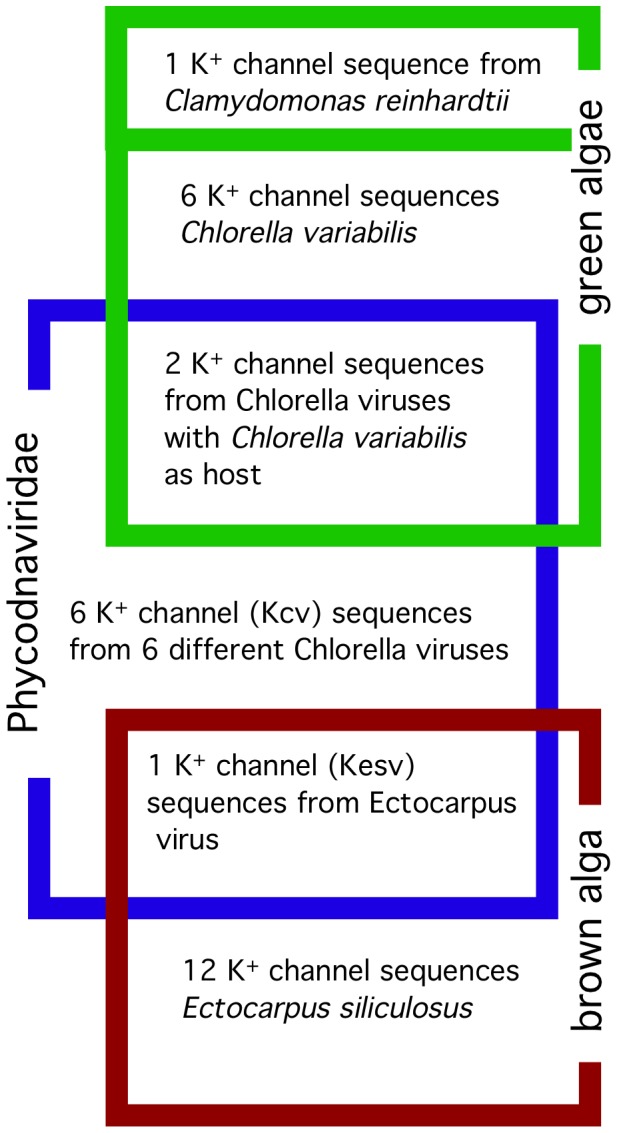
Minimal sequence set to test molecular piracy hypothesis. Seven sequences of K^+^ channels are from different phycodnaviruses. Six of them replicate in specific species of green alga*e. C. variabilis* is a host for two of these viruses. The seventh phycodnavirus infects *E. siliculosus*, a brown alga, which is only distantly related to the green algae. The viral channels are compared to putative K^+^ channels from hosts and non-hosts. The host channels include all 7 K^+^ channels from *C. variabilis* and all 12 K^+^ channels from *E. siliculosus*. A K^+^ channel sequence from the green alga *C. reinhardtii*, a non-host of phycodnaviruses and a close relative of *Chlorella* served as a negative control.

To investigate the evolution of the viral K^+^ channels and to test the ‘molecular piracy hypothesis’ in the *Phycodnaviridae*, we analyzed a small set of sequences, including host homologs, that recently became available (see Fig. 1). Our sequence data set contains seven K^+^ channels from phycodnaviruses. These viruses can be distinguished according to their host specificity. Six viruses replicate in different species of unicellular green algae. Two of these six viruses, PBCV-1 and NY-2A, specifically infect *Chlorella variabilis* (formerly *Chlorella* NC64A), two, ATCV-1 and TN603 infect *Chlorella heliozoae* (formerly *Chlorella* SAG 3.83) and two infect *Micractinium conductrix* (formerly *Chlorella* Pbi). The seventh phycodnavirus EsV-1 infects *E. siliculosus*, whose genomic information is also available [26]. The viral channels were compared to the K^+^ channels from these viral hosts and related non-host species. If gene piracy explains the presence of the viral channel genes the Kesv channel should be closely related to the EsK channels from *Ectocarpus* and Kcv_PBCV-1_ and Kcv_NY-2A_ should be close to the *C. variabilis* channels.

## Results

### Virus sequence analysis

**Figure 2:**
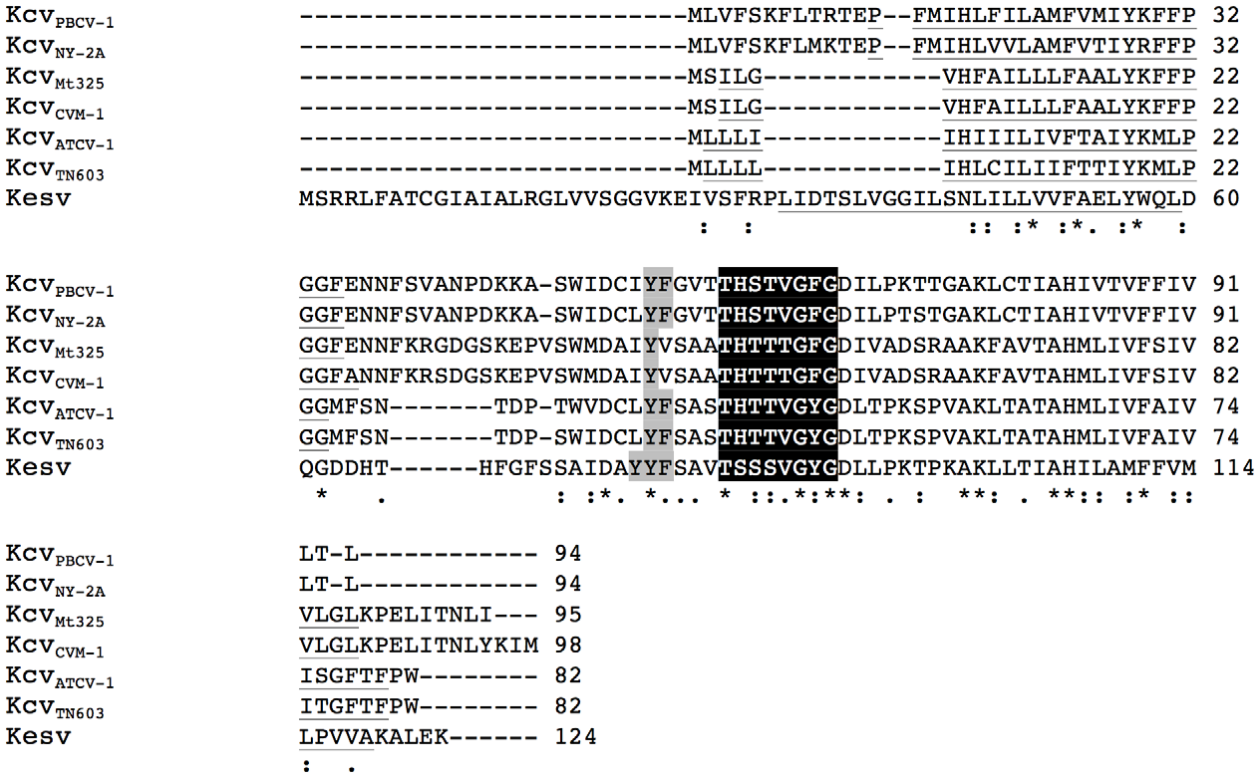
Multiple sequence alignment of K^+^ channel proteins from different phycodnaviruses. The genes that code for these proteins, originate from viruses with different host specificities. Kcv_PBCV-1_ and Kcv_NY-2A_ are from viruses that replicate in *C. variabilis*, Kcv_MT325_ and Kcv_CVM-1_ from viruses that replicate in *M. conductrix*, and Kcv_ATCV-1_ and Kcv_TN603_ from viruses that replicate in *C. heliozoae*. The channel Kesv is from virus EsV-1, which replicates in *E. siliculosus*. The selectivity filter sequence is in black; aromatic amino acids upstream of the filter are marked in grey and the transmembrane domains are underlined.

The sequences for 7 virus-encoded channel proteins are shown in Fig. 2. For six of them have already been shown to function as K^+^ channels in heterologous systems [7], [17]–[19]. The amino acid sequences of the viral K^+^ channel proteins vary among each other and this variability is apparent even within the same species. For example, a genomic analysis of 40 virus isolates from a single species, all of which replicate in *C. variabilis*, revealed that the channel proteins differed by as many as 16 amino acids from the reference channel Kcv_PBCV-1_ [19], [35]. The channel protein from *C. variabilis* virus NY-2A (Kcv_NY-2A_) is also included in the alignment in Fig. 2. Ortholog channel proteins from viruses that replicate in either *C. heliozoae* or *M. conductrix* are also each represented by two viruses.

The alignment indicates the 7 viral channel proteins have ∼23% amino acid sequence identity and 60% similarity. Notably, all of the channel proteins have the canonical selectivity filter sequence TxxTxGF/YG, which is typical for all K^+^ channel proteins from prokaryotes and eukaryotes. The 6 channels from the chloroviruses are more similar to each other than to the K^+^ channel protein from EsV-1. Hence, the diversity between the viral channels correlates with the classification of the host species.

### K^+^ channel proteins from *C. variabilis* and *E. siliculosus*

Recent sequencing of the *C. variabilis* [34] and the *E. siliculosus* genomes [26] allowed us to address the question of whether the viral K^+^ channels are more closely related to their host homologs or to each other. *C. variabilis* is the host for viruses PBCV-1 and NY-2A, while *E. siliculosus* is the host for EsV-1. We searched the two host genomes for putative K^+^-channel proteins using the following parameters:

1. All the host gene products were screened for the highly conserved motifs in the selectivity filter region (motifs: GYG, GFG and GLG), which exist in all known K^+^-channel proteins [36], [37].
2. The sequences of all known K^+^-channels and the structurally related cyclic nucleotide gated channels (CNG) from *Arabidopsis thaliana* plus additional members of other K^+^-channel families (Kir, Kv, TPA and Tandem channels from animals plus some typical microbial channels KcsA, MthK, KvAP, KvLm, KirBac1.1) were compared to the *C. variabilis* and *E. siliculosus* genomes using BLAST [38].

All genes that were detected by these methods and that had ≥2 predicted transmembrane domains were then used as queries for BLAST searches against the NCBI protein database. This search identified 7 CDSs with the hallmarks of K^+^-channels in the *C. variabilis* genome, designated CvK1-7, and 12 CDSs in the *E. siliculosus* genome, designated EsK1-12. One of them, EsK1, was nearly identical to the viral Kesv from EsV-1. However, the EsV-1 genome is incorporated into the host genome by lysogeny and so the viral channel was expected to be in the *E. siliculosus* genome [26].

**Figure 3:**
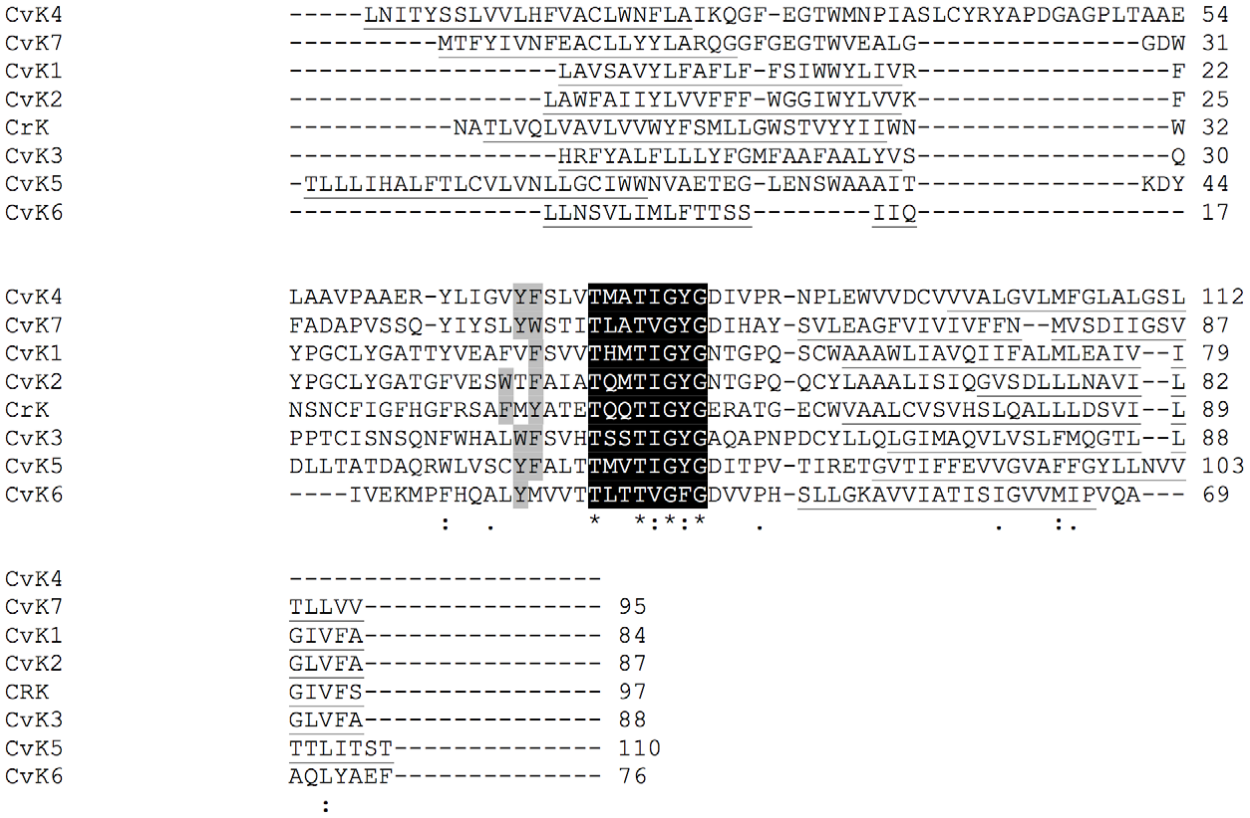
Multiple sequence alignment of pore modules of K^+^ channel proteins from *C. variabilis*. For comparison a K^+^ channel protein CRK from the alga *C. reinhardtii* is also included. The pore-forming unit begins with the transmembrane domain, prior to the selectivity filter and it finishes at the end of the transmembrane domain after the filter. The locations of transmembrane domains were predicted based on different methods. The selectivity filter sequence is in black; aromatic amino acids upstream of the filter are marked in grey; the transmembrane domains are underlined. Worth noting is the K^+^ channels conserved selectivity filter sequence and an otherwise overall low degree of similarity between the channels.

**Figure 4:**
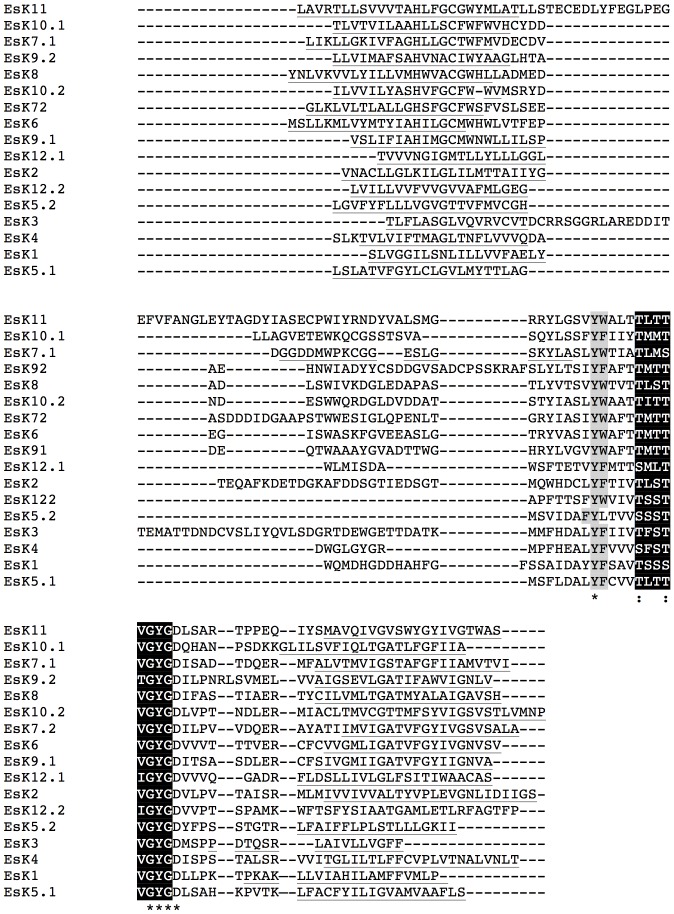
Multiple sequence alignment of pore modules of K^+^ channel proteins from *E. siliculosus*. The pore-forming unit begins with the transmembrane domain, prior to the selectivity filter and it finishes at the end of the transmembrane domain after the filter. The selectivity filter sequence is in black; aromatic amino acids upstream of the filter are marked in grey; the transmembrane domains are underlined.

We restricted our phylogenetic analyses to the pore module of these proteins, which comprises two transmembrane domains, a pore helix and the canonical selectivity filter sequence [4]. To identify the pore modules of the putative host channel proteins, all amino acid sequences were subjected to bioinformatics methods for transmembrane domain prediction (see Materials and Methods). A consensus of the predictions for the pore modules from *C. variabilis* and *E. siliculosus* are shown in Fig. 3 and Fig. 4, respectively. Several two-pore K^+^-channels were identified in *E. siliculosus*. Each pore is listed individually and the pores are indexed as x.1 or x.2 for the N- and C-terminal pores, respectively.

The alignments indicate that the pore module sequences in the *C. variabilis* and *E. siliculosus* channels are highly divergent. However, it is important to note that all the proteins have the typical architecture of K^+^ channels namely: the selectivity filter domain comprising the K^+^ channel consensus sequence and the pore helix. The latter are flanked flanked by ≥2 transmembrane domains; canonical aromatic amino acids are found upstream of the consensus sequence.

### Phylogentic analysis of K^+^ channel proteins

For a phylogenetic comparison of the viral and algal channels we only included the pore modules. A disadvantage of this analysis is that it only considers a small part of protein sequences; however this disadvantage is compensated by the fact that the pore module is the functional core domain of all K^+^ channels [4].

**Figure 5:**
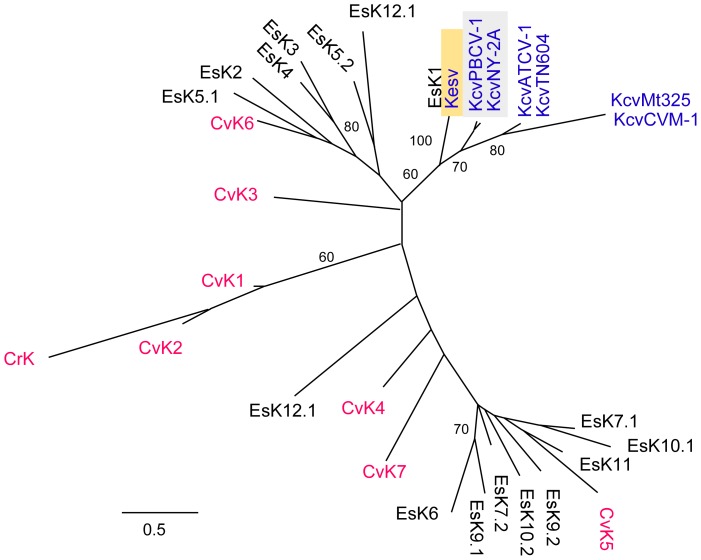
Maximum likelihood tree of K^+^ channel acid sequences from phycodnaviruses and host cells *C. variabilis and E. siliculosus*. Alignment was constructed with the use of MUSCLE [45]. The phylogenetic tree was constructed using PhyML [47] available at Phylogeny.fr [48] using WAG matrix and gamma distribution. Branch labels indicate bootstrap percentages (≥50%) after 100 replicates. The tree is essentially an unrooted tree. Viral K^+^ channels are indicated in blue, channels from green algae *C. variabilis* and *C. reinhardtii* in red. The channels from viruses, which replicate in *C. variabilis* or *E.* siliculosus are highlighted by a grey or yellow background respectively.

First we estimated the phylogenetic relationship of the channels by a maximum likelihood method. It should be noted that this analysis does not provide an in depth phylogenetic analysis of the channels. The goal was to address the question: are the viral channels descendents of host channels or do they form a separate clade? The resulting tree in Fig. 5 shows a clade containing all viral homologs that is separate from the cellular homologs, albeit with relatively low sequence similarities between viral homologs. In the tree, one of the *E. siliculosus* K^+^ channels (EsK1) was closely placed with the viral K^+^ channel Kesv. As mentioned above this result is expected since the entire genome of the lysogenic virus EsV-1 is contained in the genome of the infected host [26]. The paralogs from the two algae and CrK (*C. reinhardtii*) are more similar to each other than to the viral homologs, even though the last common ancestor between the green alga *Chlorella* and the brown alga *Ectocarpus* probably dates back more than 500 million years [27]. Apart from the similarity to EsK1, Kesv is well separated from the putative *E. siliculosus* channels. Likewise all channels from the chloroviruses form a distinct clade from the algal homologs in the tree; the channels from PBCV-1 and NY-2A, i.e., the *C. variabilis* viruses, are clearly separated from the *C. variabilis* channel sequences. The same separation of viral channels from the host channels was also observed in trees produced with a parsimony method and a neighbor-joining algorithm (Figs S1, S2).

**Figure 6:**
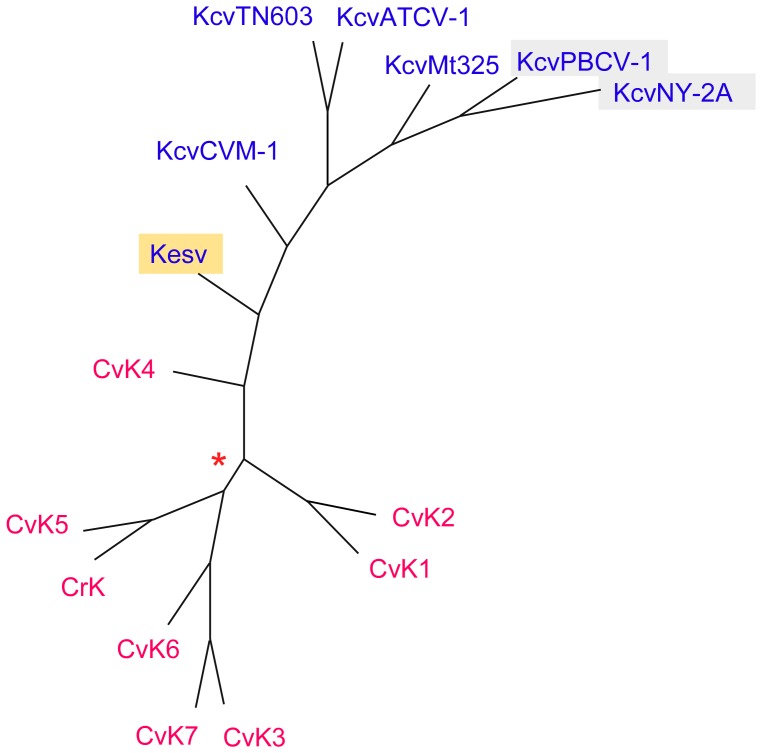
Consensus, unrooted tree obtained by Bayesian estimates of phylogenies for the amino acid and nucleotide sequences, as well as for a protein parsimony approach. All clades showed a statistical support of 1 (=100%) with reference to the six independent trees computed (Bayesian estimate). The same holds for the statistical support with reference to the 1,000 replicas fed into the protpars program (protein parsimony). The branch length in this tree is arbitrary. The only difference between these is a weaker support in one of the clades (50% support, as indicated by the red star). Note that all phylogenetic approaches resulted in the same tree. Red entries indicate algae channels, while blue entries are viral channels.

Next we analyzed the phylogenetic relationship of the channels from *C. variabilis* and viruses using Bayesian estimates. Fig. 6 shows the consensus tree of phylogenies obtained by Bayesian estimation from nucleotide and amino acid sequences, as well as by a protein maximum parsimony method. This analysis indicated that the viral channels form a clade, which is clearly separate from the second clade containing the algal channels. A K^+^ channel (CrK) from another unicellular green alga, *C. reinhardtii*, grouped with a homolog from *C. variabilis*. The clear separation between the viral K^+^ channels and the algal channels occurs even when the amino acid or nucleotide sequences were analyzed individually; this separation is evident in spite of the large diversity on the nucleotide level (Fig. S3). Furthermore, the same results are obtained using different statistical methods (see Materials and Methods).

Collectively, these data suggest that the viral channels have a long evolutionary history, which is independent from their hosts (i.e. gene duplications) and also from the deep host speciation events (i.e. *Chlorella/Chlamydomonas* and green/brown algae divergences).

### Search for the ancestor of the viral K^+^ channels

**Figure 7:**
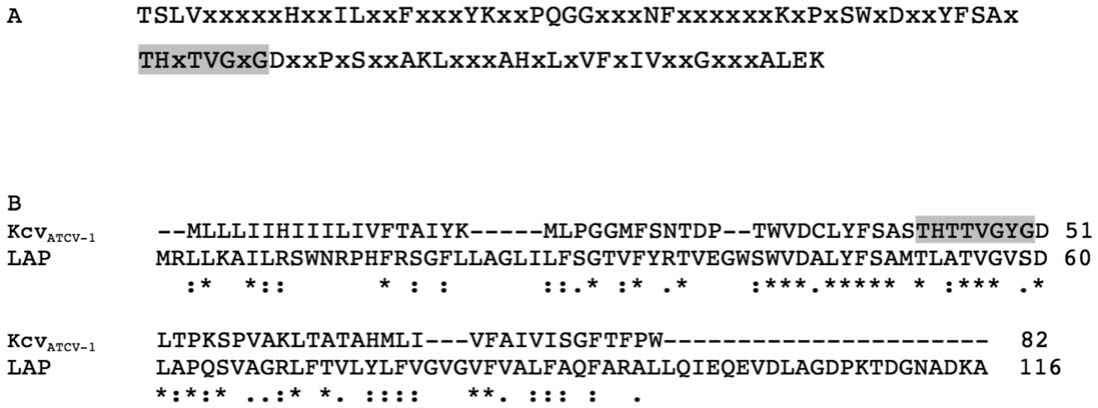
The consensus sequence of viral K^+^ channel pore is similar to protein LAP from proteobacterium *Labrenzia alexandrii DFL-11*. (A) Consensus sequence of viral K^+^ channels. (B) Alignment of K^+^ channel Kcv_ATCV-1_ with protein LAP from *L. alexandrii* DFL-11 (data bank ZP_05113853). Identical amino acids are indicated by (*), conserved or semi-conserved amino acids are indicated by (:) and (.) respectively. Note that the consensus sequence of K^+^ channel selectivity filter (grey box) is only partially conserved in the bacterial protein.

The fact that all of the viral K^+^ channel proteins group together in a common clade prompted us to identify a consensus sequence (Fig. 7A) from the viral channels using the standard procedure in the Biopython software (http://biopython.org/wiki/Main_Page) that could be used in a BLAST search to hunt for similar channel proteins. The search resulted in one hit, albeit with only moderate significance, to a protein (labeled LPA) from the marine proteobacterium *Labrenzia alexandrii* DFL-11 (GenBank: NZ_EQ973121).

Fig. 7B shows an alignment of LPA from *L. alexandrii DFL-11* and Kcv_ATCV_, the viral channel that is most similar to LPA. The alignment reveals many identical or similar residues in the transmembrane domains. However, LPA from *L. alexandrii DFL-11* lacks the canonical sequence of K^+^ channels [36], [37] and probably does not function as a K^+^ channel.

**Figure 8:**
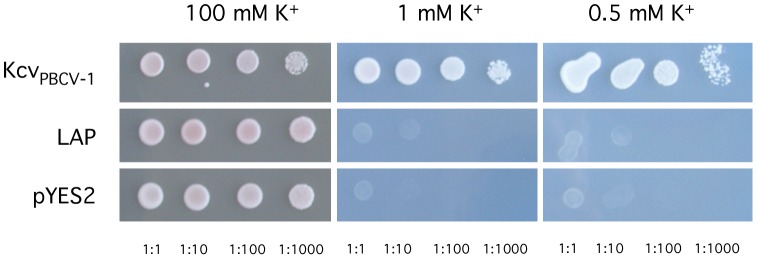
Growth phenotype ΔtrkΔtrk2 mutants transformed with different genes. Yeast cells were transformed with either an empty vector or with genes encoding viral K^+^ channel Kcv, or the protein LAP from *L. alexandrii DFL-11*. All yeasts were grown on non-selective medium containing either 100 mM K^+^ or lesser amounts. Only yeast transformed with Kcv_PBCV-1_ grew on selective medium with low 0.5 mM and 1 mM K^+^ concentrations.

We tested the functionality of LPA as a K^+^ channel by cloning and expressing its gene in mutants of yeast that are devoid of K^+^ uptake systems. These mutants only grow in a medium with high K^+^ (100 mM). Growth on a medium with low K^+^ can only occur by expressing a heterologous K^+^ channel [29]. The data in Fig. 8 show that all yeast mutants grow on medium with high K^+^. Growth on medium with low K^+^ only occurred when cells are transformed with the functional Kcv_PBCV-1_. This result is consistent with the previous observation that functional viral K^+^ channels can rescue the yeast mutants under selective conditions [29]. However, LPA from *L. alexandrii DFL-11* did not rescue the mutant defect. Although this result does not provide definitive proof that LPA is not a K^+^ channel protein, it indicates that it probably does not form a functional channel in yeast. Together, these results indicate it is unlikely that the viral channels came from proteobacteria.

## Discussion

The viral K^+^ channels are small and basically consist of the pore module shared by all K^+^ channels [4], [10]. Our analyses indicate that viral-encoded K^+^ channel proteins do not have a close phylogenetic relationship with their host-encoded K^+^ channel proteins. A similar scenario was recently discovered for a chlorovirus encoded cation transporter which occurs in different virus species independent of their host [39]. The diversity of the phylogenetic methods used in this study, which produced similar results, reduces the likelihood that our conclusions are due to phylogenetic artifacts. Different phylogenetic approaches also indicate that the viral K^+^ channels are relatively closely related to each other in spite of the large sequence divergence between some of the virus gene pairs [23]. Clearly, our results contradict the ‘molecular piracy hypothesis’ where viral genes are assumed to be transferred from their hosts; thus alternative explanations are required. The present data are consistent with two possibilities. First, the viral genes were recently transferred from unidentified hosts of the viruses. Given the remarkable diversity of K^+^ channel proteins in eukaryotes and their underrepresentation in the current sequence databases, eukaryotic homologs similar to the viral channel genes may be found in the future. Based on the large distances between the viral and host homologs in our phylogenetic trees, we predict that such unidentified hosts would be distantly related to *Chlorella* and *Ectocarpus*. This explanation is a modified version of the ‘molecular piracy hypothesis’. This scenario, however, requires a drastic recent change of host types (or a capacity to infect totally different hosts) for these viruses. Another scenario is that viral channel homologs evolved prior to or at the time of the divergence of eukaryotic algae. The viral K^+^ channels could arise from an ancient cellular organism, which served as a host for these viruses before they developed their present host specificity, or they might directly originate from the ancient virus world. The latter hypothesis of a viral origin for K^+^ channels is not that surprising, when one considers that many other viruses code for very simple and viral specific proteins with ion channel functions [1]–[4], [40]. In these examples, some or all K^+^ channels in cellular organisms might be derived from ancestral viral proteins. Since viruses typically have high mutation and recombination rates as well as very high reproduction rates relative to their hosts, a relaxed selection due to complementation, for instance, may be an evolutionary mechanism that enhances the creation of new genes in viral genomes. This is consistent with the occurrence of many small genes of unknown function in viral genomes (ORFans) [41], [42] including numerous small membrane proteins [43]. The functions of the viral channels with respect to their pharmacology and voltage dependency can be quite different [17]–[19]. The forces, which determine this structural and functional diversity of channel proteins among viruses, may be assigned to virus-virus competition. The activity of the channels from the chloroviruses is probably essential for infection and in a later step also important in preventing hyper-infection [12]–[14]. Since the viral channels presumably contribute to depolarization of the host plasma membrane and since some virus species seem to out-compete others in an experimental setting by the speed with which they depolarize their host [12], it is reasonable to assume that this competition is a driving force for channel diversification. The fact, that the virus EsV-1 channel is associated with the mitochondria [29], suggests that this channel protein acquired domains that sort the protein to this organelle. The competition for the right molecular sorting machinery must have affected the evolution of this protein. Since the EsV-1 protein is in the mitochondria, this channel might be part of an early anti-apoptotic system important for viral persistence.

## Materials and Methods

### Sequences

Six Kcv type K^+^ channel proteins from chloroviruses PBCV-1, NY-2A, MT325, CVM-1, ATCV-1, TN603, and one Kesv channel from virus EsV-1 were analyzed. We also identified 7 K^+^ channel protein sequences in *C. variabilis* (see results), the host for viruses PBCV-1 and NY-2A and 12 K^+^ channel proteins in *E. siliculosus*. The sequence of a putative K^+^ channel from the non-host green alga *Chlamydomonas reinhardtii* was also included in the analyses. Sources for the genes are provided in Table S1. With this set of channels we derived a set of sequences to test the molecular piracy hypothesis.

The sequences were aligned with CLUSTALW2 [44] and/or Muscle [45], using standard parameters that produced a seed file for all further phylogenetic computations.

### Sequence analysis

Four independent approaches were used in the phylogenetic experiment:

1. Maximum likelihood estimation.
2. Bayesian estimation of phylogeny from the nucleotide sequences.
3. Bayesian estimation of phylogeny from the translated amino acid sequences.
4. Protein Sequence Parsimony Methods as implemented in protpars of the phylip package [46] were applied to the translated amino acid sequences.

For the maximum likelihood estimates we used PhyML [47] available at Phylogeny.fr [48] using WAG matrix and gamma distribution as default parameters. For the derivation of amino acid phylogenies by Bayesian estimation we used the MrBayes package [49], [50] with default parameters. We made six independent trees with 70,100,000 iterations each. Here the standard deviation of split frequencies reached 10^−3^. For the final trees we obtained a consensus tree by the consensus program of the phylip package Version 3.67 [46].

For the Bayesian estimation of phylogeny of the nucleotide sequences we again used MrBayes, and reached good convergence after 3,600,000 iterations with the same convergence limit as for the protein sequences. We performed five independent runs and computed a consensus tree as above. The protein parsimony was performed on 1,000 randomized replicas by the protpars program of Phylip. Randomization was done by the internal routine of the protpars program. From the resulting 1,000 trees we computed a consensus tree as above. The consensus sequence of the viral K^+^ channels was obtained from an alignment of the pore modules of these channels and calculated with a tool in the Biophyton software. The pore module comprises the amino acid sequences from the beginning of the first transmembrane domain to the end of the second transmembrane domain. The pore model of all channels was identified from the primary amino acid sequences using the following transmembrane region prediction algorithms: DAS, HMMTOP, SOSUI, TMpred, TMHMM, TopPred, MPEx. We used a consensus result for the prediction of the TMDs.

### *Saccharomyces cerevisiae* complementation assays

Selection experiments were performed as reported previously [29]. Viral K^+^ channel encoding genes or their mutants were transformed into SGY1528 yeast strain (*Mat a ade*2–1 *can*1–100 *his*3–11,15 *leu*2–3,112 *trp*1–1 *ura*3–1 *trk*1::HIS3 *trk*2::TRP1), which is deficient in endogenous K^+^ uptake systems. Yeasts from the same stock were grown in parallel under nonselective conditions on plates containing 100 mM KCl and on selective conditions on agar containing 1 mM KCl or 0.5 mM KCl. Growth experiments were conducted at 30°C.

## Supporting Information

Table S1 **Information on source of K^+^ genes.**
(DOC)

Figure S1 **Parsimony tree of K^+^ channel amino acid sequences from phycodnaviridae and host cells** ***C. variabilis and E. siliculosus*** **.**
(DOC)

Figure S2 **Neighbor joining tree of K^+^ channel amino acid sequences from phycodnaviridae and host cells** ***C. variabilis and E. siliculosus*** **.**
(DOC)

Figure S3 **Multiple sequence alignment of nucleotides coding for K^+^ channel pores from in** ***C. variabilis, C. reinhardtii and phycodnaviridae*** **.**
(DOCX)
