# Supplementary material for: Phycodnavirus Potassium Ion Channel Proteins Question the Virus Molecular Piracy Hypothesis
Source: PLoS One. 2012 Jun 7;7(6):e38826. doi: 10.1371/journal.pone.0038826 (PMC3369850; doi:10.1371/journal.pone.0038826)
Supplement: Table S1 — Information on source of K+ genes. (DOC) [file pone.0038826.s001.doc]

**Table S1:** Information on K^+^ channel sequences used for the analysis. Sequences of listed channels (GenBank number in round brackets, CDS numbers in square brackets) were obtained from NCBI for *Chlamydomonas reinhardtii* channel CRK and for virus channels Kcv_PBCV-1_, Kcv_NY-2A_ , Kcv_Mt325_ , Kcv_ATCV-1_ and Kesv. Additional sequences for Kcv_CVM-1_ and Kcv_TN603_ were obtained from a *Chlorella* virus database (<http://greengene.uml.edu/> database/database.htm). *Chlorella* NC64A channels were obtained from DOE joint genome institute (JGI) at (<http://genome.jgi-psf.org/cgi-bin/ToGo?species=ChlNC64A_1>) and *Ectocarpus siliculosus* channels at (http://bioinformatics.psb.ugent.be/genomes/view/Ectocarpus-siliculosus).

| **channel** | **(GenBank number)/**  **[CDS number]** | **Organism** |
| --- | --- | --- |
| Kcv_PBCV-1_ | (AAQ16129)/[A250L] | PBCV-1 |
| Kcv_NY-2A_ | (YP_001497532)/[B336R] | Chlorella virus NY-2A |
| Kcv_Mt325_ | (ABT13737)/[M183R] | Chlorella virus Mt325 |
| Kcv_ATCV-1_ | (YP_001427066)/[Z585R] | Chlorella virus ATCV-1 |
| Kesv | (NP_077708 08)/[EsV-223]. | EsV-1 |
| Kcv_CVM-1_ | [P59_023] | *Chlorella virus CVM-1* |
| Kcv_TN603_ | [Y02_007R] | *Chlorella virus TN603* |
| CvK1 | (IGS.gm_1_00193) | *Chlorella variabilis* |
| CvK2 | (estExt.fgenesh3.pg.C_40067) | *Chlorella variabilis* |
| CvK3 | (IGS.gm_20_00009) | *Chlorella variabilis* |
| CvK4 | (fgenesh3_pg.C_scaffold_9000225) | *Chlorella variabilis* |
| CvK5 | (IGS.gm_7_00033) | *Chlorella variabilis* |
| CvK6 | (fgenesh3_pg.C_scaffold_8000194) | *Chlorella variabilis* |
| CvK7 | (IGS.gm_18_00217) | *Chlorella variabilis* |
| CrK | (XP_001691185.1) | *Chlamydomonas reinhardtii* |
| EsK1 | ([Esi0052_0061](http://bioinformatics.psb.ugent.be/webtools/bogas/annotation/Ectsi/current/Esi0052_0061)) | *Ectocarpus siliculosus* |
| EsK2 | ([Esi0323_0020](http://bioinformatics.psb.ugent.be/webtools/bogas/annotation/Ectsi/current/Esi0323_0020)) | *Ectocarpus siliculosus* |
| EsK3 | ([Esi0056_0024](http://bioinformatics.psb.ugent.be/webtools/bogas/annotation/Ectsi/current/Esi0056_0024)) | *Ectocarpus siliculosus* |
| EsK4 | ([Esi0217_0029](http://bioinformatics.psb.ugent.be/webtools/bogas/annotation/Ectsi/current/Esi0217_0029)) | *Ectocarpus siliculosus* |
| EsK5 | ([Esi0177_0044](http://bioinformatics.psb.ugent.be/webtools/bogas/annotation/Ectsi/current/Esi0177_0044)) | *Ectocarpus siliculosus* |
| EsK6 | ([Esi0000_0275](http://bioinformatics.psb.ugent.be/webtools/bogas/annotation/Ectsi/current/Esi0000_0275)) | *Ectocarpus siliculosus* |
| EsK7 | ([Esi0271_0023](http://bioinformatics.psb.ugent.be/webtools/bogas/annotation/Ectsi/current/Esi0271_0023)) | *Ectocarpus siliculosus* |
| EsK8 | ([Esi0180_0012](http://bioinformatics.psb.ugent.be/webtools/bogas/annotation/Ectsi/current/Esi0180_0012)) | *Ectocarpus siliculosus* |
| EsK9 | ([Esi0015_0103](http://bioinformatics.psb.ugent.be/webtools/bogas/annotation/Ectsi/current/Esi0015_0103)) | *Ectocarpus siliculosus* |
| EsK10 | (Esi0127_003) | *Ectocarpus siliculosus* |
| EsK11 | (Esi0155_0068) | *Ectocarpus siliculosus* |
| EsK12 | (Esi0084_0073) | *Ectocarpus siliculosus* |
