# Supplementary material for: Phycodnavirus Potassium Ion Channel Proteins Question the Virus Molecular Piracy Hypothesis
Source: PLoS One. 2012 Jun 7;7(6):e38826. doi: 10.1371/journal.pone.0038826 (PMC3369850; doi:10.1371/journal.pone.0038826)
Supplement: Figure S1 — Parsimony tree of K+ channel amino acid sequences from phycodnaviridae and host cells C. variabilis and E. siliculosus . (DOC) [file pone.0038826.s002.doc]

Fig. S1


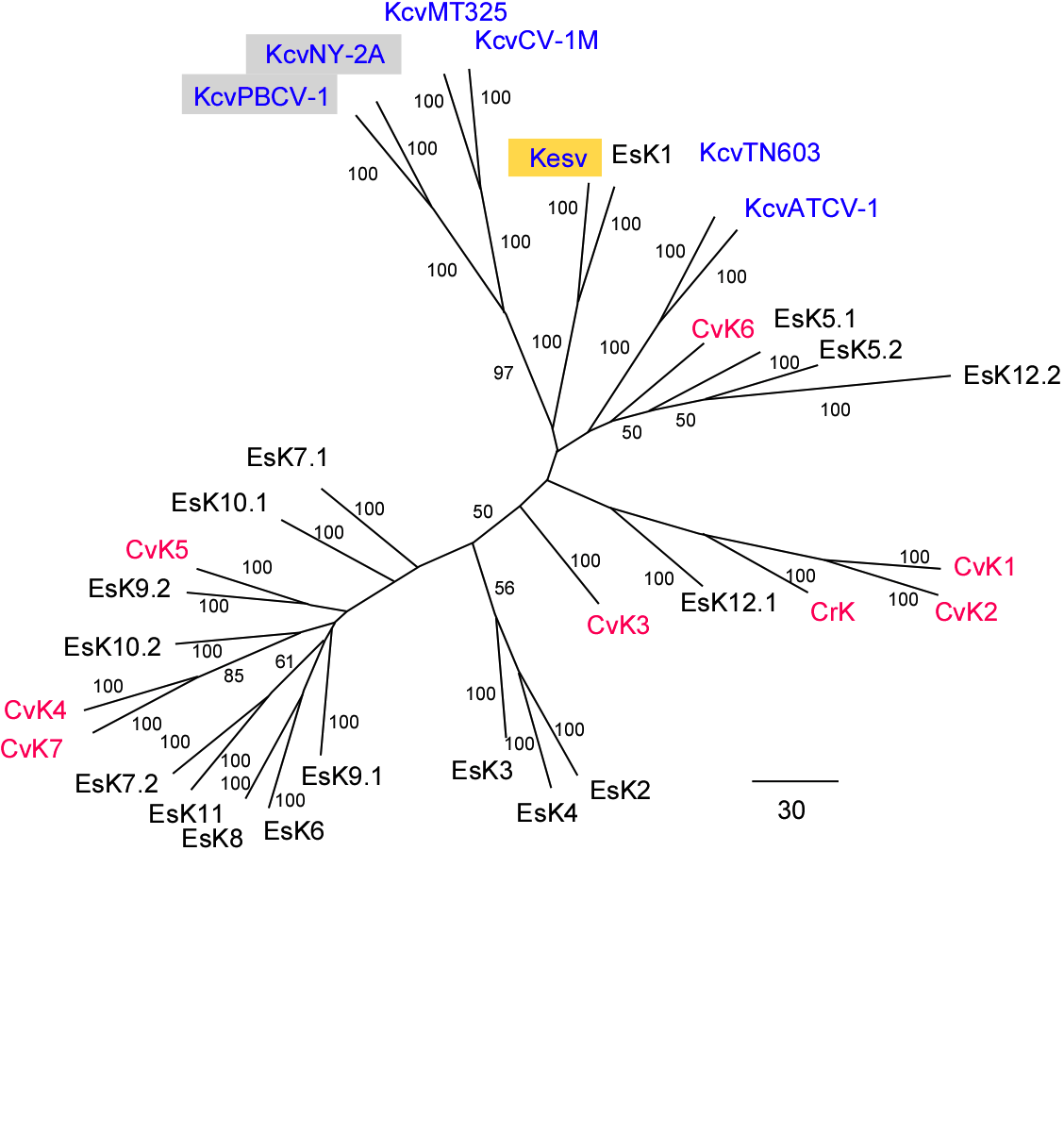


**Parsimony tree of K^+^ channel amino acid sequences from phycodnaviridae and host cells *C. variabilis and E. siliculosus***. Alignment was constructed with the use of MUSCLE (Edgar & Robert, 2004). The phylogenetic tree was constructed using protpars program of Phylip (http://mobyle.pasteur.fr) using standard settings. Branch labels indicate bootstrap percentages (≥ 50%) after 100 replicates. Tree topology and bootstrap values were similar when using different alignment programs. The tree is essentially an un-rooted. Viral K^+^ channels are indicated in blue, channels from green algae Chlorella NC64 A and *Chlamydomonas reinhardtii* in red. The channels from viruses, which replicate in *C. variabilis* or *E.* siliculosus are highlighted by a grey or yellow background respectively.
