# Supplementary material for: Phycodnavirus Potassium Ion Channel Proteins Question the Virus Molecular Piracy Hypothesis
Source: PLoS One. 2012 Jun 7;7(6):e38826. doi: 10.1371/journal.pone.0038826 (PMC3369850; doi:10.1371/journal.pone.0038826)
Supplement: Figure S3 — Multiple sequence alignment of nucleotides coding for K+ channel pores from in C. variabilis, C. reinhardtii and phycodnaviridae . (DOCX) [file pone.0038826.s004.docx]

Fig. S3

CvK1 ------------------------------------------------------------

CvK2 ------------------------------------------------------------

CvK5 ---------------------------ACCCTGCTGCTCATCCACGCCCTCTTCACCCTC

CrK ---------------------------------------------------------AAC

KcvPBCV-1 ------------------------------------------------------------

KcvNY-2A ------------------------------------------------------------

KcvMt325 ------------------------------------------------------------

KcvCVM-1 ------------------------------------------------------------

KcvATCV-1 ------------------------------------------------------------

KcvTN603 ------------------------------------------------------------

Kesv ------------------------------------------------------------

CvK3 ------------------------------------------------------------

CvK6 ------------------------------------------------------------

CvK4 CTCAACATCACCTACTCCTCCCTCGTGGTCCTCCACTTCGTGGCCTGTCTCTGGAACTTC

CvK7 ---------------------------------------------------------ATG

CvK1 ------------------CTGGCTGTCTCCGCGGTG---TACCTCTTTGCCTTCCTCTTC

CvK2 ------------------CTGGCCTGGTTTGCGATCATCTACCTGGTCGTCTTCTTCTTC

CvK5 TGCGTGCTGGTCAACCTGCTGG-GCTGCATTTGGTGGAACGTGGCGG-AGACTGAGGGGC

CrK GCCACGCTGGTCCAGCTGGTGGCGGTGCTGGTGGTGTGGTACTTCTCTATGCTACTGGGC

KcvPBCV-1 ------------CCATTCATGATACATCTCTTTATTCTCGCAATGTTCGTGATGATCTAT

KcvNY-2A ------------CCATTCATGATTCATCTCGTGGTTCTCGCGATGTTCGTCACGATCTAT

KcvMt325 ------------ATTCTAGGAGTTCATTTTGCCATTTTGCTGTTATTTGCCGCATTATAC

KcvCVM-1 ---------------CTAGGAGTTCATTTTGCCATTTTGCTGTTATTTGCCGCATTATAC

KcvATCV-1 ----------------------TACATATCATCATTCTGATAGTGTTCACTGCCATCTAC

KcvTN603 ---------TTGCTGCTTCTCATACACCTCTGTATTTTGATAATTTTTACTACAATATAC

Kesv --------ACTTCGCTCGTCGGCGGAATATTGTCTAATCTGATTTTGCTCGTCGTTTTCG

CvK3 ----------------CACCGCTTCTACGCACTCTTC-CTGCTCCTCTACTTTGGCATGT

CvK6 ---------------------------------------------------CTCCTCAAC

CvK4 CTGGCCATCAAGCAAGGCTTCGAAGGCACCTGGATGAACCCCATAGCCTCCCTCTGCTAC

CvK7 ACTTTCTACATCGTCAACTTTGAGGCCTGCCTGCTCTACTACCTGGCCAGGCAGGGAGGC

CvK1 TTCTCCATC-TGGTGGTATC----TCATCGTCAGGTTCTACCCGGGCTGCCTATATGGCG

CvK2 TGGGGCGGC-ATCTGGTACC----TGGTGGTGAAGTTCTACCCCGGCTGCCTGTACGGCG

CvK5 TGGAGAACTCGTGGGCTGCCGCCATCACCAAGGATTACGACTTGCTCACCGCCACCGACG

CrK TGGAGCACT-GTGTACTAC----ATCATCTGGAACTGGAACTCCAACTGCTTCATCGGCT

KcvPBCV1 AAATTCTTCCCGGGAGGGTT--CGAAAATAACTTCTCTGTTGCAAACCCGGACAAAAAG-

KcvNY-2A CGTTTCTTCCCTGGCGGATT--CGAAAATAACTTTTCTGTTGCAAATCCGGACAAAAAG-

KcvMt325 AAATTCTTCCCAGGCGGATT--TGAGAACAATTTCAAACGCGGAGATGGGTCCAAAGAAC

KcvCVM-1 AAATTCTTCCCGGGCGGATT--CGCAAACAATTTCAAACGCAGTGATGGATCCAAAGAAC

KcvATCV-1 AAGATGCTCCCCGGCGG-----------CATGTTCTCGAACACAGACC------------

KcvTN603 AAGATGTTGCCCGGAGG-----------CATGTTCTCGAACACGGACC------------

Kesv CTGAACTTTATTGGCAGCTG-----GACCAAGGGGATGATCACACACA----CTTCGGCT

CvK3 TTGCGGCCT-TTGCGGCGCT-----GTACGTCAGCCAGCCCCCCACCTGCATCTCCAACT

CvK6 TCCGTGCTCATCATGCTCTT--------CACCACCTCCTCCATCATCCAGATCGTGGAGA

CvK4 CGCTACGCGCCCGACGGAGCCGGCCCGCTGACTGCGGCGGAGCTGGCGGCGGTGCCGGCG

CvK7 TTCGGGGAGGGCACCTGGGTGGAGGCGCTGGGCGGCGACTGGTTTGCCGACGCGCCAGTG

CvK1 -CCACCACGTACGTGGAAGCCTTTGTGTTTTCTGTGGTCACTCACATGACCATTGGCTAC

CvK2 -CCACGGGCTTTGTGGAGAGCTGGACCTTTGCCATCGCAACCCAGATGACCATCGGCTAC

CvK5 -CCCAGCGCTGGCTCGTCTCCTGCTACTTCGCCCTGACCACCATGGTCACGATAGGCTAC

CrK -TCCACGGCTTCCGCTCCGCCTTCATGTACGCCACGGAGACACAGCAGACCATTGGCTAC

KcvPBCV-1 ---GCATCATGGATAGATTGTATATACTTCGGAGTAACGACACACTCTACTGTCGGATTC

KcvNY-2A ---GCATCATGGATAGATTGTTTATATTTCGGAGTAACGACACACTCGACCGTCGGGTTC

KcvMt325 -CTGTCTCATGGATGGATGCGATATATGTCTCGGCCGCCACACACACAACAACAGGTTTT

KcvCVM-1 -CTGTATCATGGATGGATGCGATATATGTTTCTGCAGCCACACACACAACAACAGGTTTT

KcvATCV-1 ----CTACTTGGGTTGATTGCCTGTACTTTTCGGCATCGACGCACACCACCGTGGGGTAC

KcvTN603 ----CGTCATGGATAGATTGCCTGTACTTCTCGGCATCAACGCACACCACCGTGGGGTAC

Kesv -TCTCGTCCGCGATCGACGCTTACTACTTCAGTGCGGTCACGTCTTCCTCTGTCGGATAC

CvK3 -CACAAAACTTCTGGCACGCCCTCTGGTTCAGCGTGCACACCTCCAGCACCATAGGATAT

CvK6 -AGATGCCCTTCCACCAGGCGCTCTACATGGTGGTGACCACGCTGACGACCGTGGGTTTT

CvK4 GCGGAGCGGTACCTGATCGGCGTATACTTCAGTCTCGTCACGATGGCCACCATTGGGTAT

CvK7 TCCTCGCAGTACATCTACTCCCTCTACTGGTCTACCATCACGCTCGCGACCGTGGGATAT

** * ** *

CvK1 GGCAACACAGGGCCGCAGT-CGTGCTG--GGCTGC-TGCGTGGCTGATTGCGGTGCAGAT

CvK2 GGCAACACGGGGCCCCAGC-AGTGCTA--CCTGGC-GGCTGCGCTGATCTCCATCCAGGG

CvK5 GGCGACATCACTCCGGTCA-CAATCAG--GGAGACCGGCGTCACCATCTTCTTTGAGGTG

CrK GGCGAGCGCGCCACTGGCG-AGTGCTG--GGTGGC-GGCGCTGTGTGTGTCCGTGCACTC

KcvPBCV-1 GGAGATATACTGCCAAAGA-CGACCGG--CGCAAAGCTTTGTACGATAGCACATATAGTA

KcvNY-2A GGAGATATACTACCTACTT-CGACCGG--CGCGAAACTTTGTACGATCGCACACATTGTC

KcvMt325 GGAGATATTGTGGCGGACT-CAAGAGC--TGCGAAGTTTGCAGTGACGGCTCACATGTTG

KcvCVM-1 GGAGATATTGTGGCAGACT-CAAGAGC--CGCGAAGTTTGCAGTCACGGCTCATATGTTG

KcvATCV-1 GGAGATCTCACGCCCAAAT-CACCCGT--GGCAAAACTCACGGCAACGGCACACATGTTG

KcvTN603 GGGGATCTCACGCCCAAAT-CGCCCGT--GGCAAAACTCACAGCAACGGCACACATGCTG

Kesv GGCGATTTGTTGCCGAAAA-CTCCGAA--GGCAAAATTGCTTACCATCGCACACATTTTG

CvK3 GGCGCCCAGGCGCCCAACC-CGGACTGCTACCTGCTCCAGCTGGGCATCATGGCGCAGGT

CvK6 GGCGACGTGGTGCCCCACAGCCTGCTGGGCAAGGCGGTGGTCATCGCCACCATCTCCATC

CvK4 GGCGACATCGTGCCCAGGAACCCGCTTGAGTGGGTGGTGGACTGCGTGGTGGTGGCCCTG

CvK7 GGCGACATCCATGCCTATAGCGTCCTGGAGGCGGGCTTTGTTATTGTCATCGTCTTTTTC

** *

CvK1 CATCTTCGCTCTGATGCTAG--AGGCGATTGTCATTGGCATCGTATTTGCC---------

CvK2 CGTCTCCGACCTGCTGCTGA--ACGCAGTCATCCTGGGCCTCGTCTTTGCC---------

CvK5 GTGGGAGTCGCCTTCTTCGGCTACCTCCTGAATGTGGTCACCACCCTGATCACCTCAACC

CrK CCTACAGGCGCTGCTGCTGG--ACAGCGTCATCCTGGGCATTGTGTTCAGC---------

KcvPBCV-1 ACAGTGTTCTTCATCGTTCTAACT------------------------------------

KcvNY-2A ACGGTATTTTTCATCGTG------------------------------------------

KcvMt325 ATAGTGTTTTCGATCGTTGTTCTAGGATTGAAG---------------------------

KcvCVM-1 ATAGTGTTTTCGATCGTTGTTCTAGGATTG------------------------------

KcvATCV-1 ATCGTATTCGCGATCGTCATTTCTGGCTTCACGTTT------------------------

KcvTN603 ATCGTATTCGCGATCGTAATAACTGGCTTCACATTCCCG---------------------

Kesv GCCATGTTCTTCGTGATGCTCCCCGTTGTCGCGAAGGCTCTCGAAAAG------------

CvK3 GCTGGTCAGCCTGTTCATGC--AGGGCACGCTGCTGGGCCTGGTCTTTGCC---------

CvK6 GGCGTGGTCATGATCCCGGTGCAGGCGGCCCAGCTGTACGCAGAGTTC------------

CvK4 GGGGTGCTCATGTTTGGCCTGGCGCTGGGCAGCCTG------------------------

CvK7 AATATGGTGAGCGACATCATCGGTAGCGTCACGCTGCTGGTCGTG---------------

Multiple sequence alignment of nucleotides coding for pore modules of K^+^ channel proteins from *C. variabilis*, *C. reinhardtii* and from *phycodnaviridae*. The pore-forming unit begins with the transmembrane domain, prior to the selectivity filter and it finishes at the end of the transmembrane domain after the filter. Worth noting is the K^+^ channels conserved selectivity filter sequence and an otherwise overall low degree of similarity between the channels. Alignment was performed with CLUSTAL 2.0.9.
